# Longitudinal changes in home food availability and concurrent associations with food and nutrient intake among children at 24–48 months

**DOI:** 10.1017/S1368980024000375

**Published:** 2024-02-02

**Authors:** Jennifer M Barton, Arden L McMath, Stewart P Montgomery, Sharon M Donovan, Barbara H Fiese

**Affiliations:** 1 Family Resiliency Center, University of Illinois, Urbana-Champaign, Urbana, IL, USA; 2 Center for Childhood Obesity Research, Pennsylvania State University, University Park, PA, USA; 3 Division of Nutritional Sciences, University of Illinois, Urbana-Champaign, Urbana, IL, USA; 4 Department of Food Science and Human Nutrition, University of Illinois, Urbana-Champaign, Urbana, IL, USA; 5 Department of Human Development and Family Studies, University of Illinois, Urbana-Champaign, Urbana, IL, USA

**Keywords:** STRONG Kids 2, Home food availability, FFQ, Early childhood

## Abstract

**Objectives:**

To describe changes in home food availability during early childhood, including modified, developmentally sensitive obesogenic scores, and to determine whether home food availability is associated with food and nutrient intakes of children concurrently, over time.

**Design:**

Data were drawn from the STRONG Kids 2 longitudinal, birth cohort to achieve the study objectives. Home food availability was assessed with the Home Food Inventory (HFI) and included fifteen food groups (e.g. fruit and vegetables) and three obesogenic scores (one original and two modified). Food and nutrient intakes were measured using the Block FFQ and included twenty-seven food groups and eighteen nutrients (e.g. vitamins A and C, protein). HFI and FFQ were completed by trained researchers or mothers, respectively, at 24, 36 and 48 months. Repeated-measures ANOVA and Spearman’s correlations were used to achieve the study objectives.

**Setting:**

Central Illinois, USA.

**Participants:**

Participants were 468 children at 24, 36 and 48 months of age.

**Results:**

Availability of less nutritious foods and obesogenic foods and beverages increased as children aged, and availability of both nutritious and less nutritious foods were associated with child food and nutrient intake. The three obesogenic scores demonstrated similar, positive associations with the intake of energy, saturated fat, added sugars and kilocalories from sweets.

**Conclusion:**

These findings offer novel insight into changes in home food availability and associations with food and nutrient intake during early childhood. Additional attention is needed examining antecedents (e.g. built environments, purchasing behaviours) and consequences (e.g. child diet quality and weight) of home food availability.

Early childhood is an important period for learning about nutrition and for gaining greater autonomy in the decision-making of their food preferences^(1)^. Parents are responsible for selecting which foods and beverages to purchase and offer to their children, producing the home food environment. The home food environment includes physical and sociocultural characteristics, such as food availability and parents’ feeding practices^(2)^. Home food availability is defined as the presence of food items on countertops, in refrigerators, or in pantries^(3)^ and contributes to children’s food choices and preferences^(4)^. These preferences can have long-term consequences for child health, likely through their dietary patterns^(5,6)^. For example, children exposed to more processed foods may also have decreased exposure to fruits and vegetables^(7)^. This lack of exposure, or preference development, may lead to decreased consumption into adolescence and adulthood, placing the child at risk for nutrient deficiencies and adverse chronic health conditions such as overweight and obesity, hypertension, and diabetes^(8–10)^. Considering that home food availability has the capacity to influence child diet and health, there is limited research examining the validity of food inventories with early childhood samples.

Home food availability has often been measured using inventories, or checklists, of available foods and beverages^(11)^. Earlier inventories were created to address specific food items (e.g. fruit and vegetables, high-fat *v*. low-fat foods) for use in disease prevention studies, and thus, were not designed to provide a comprehensive account of foods available. Another limitation of earlier inventories is reliance on self-report, which could introduce additional bias^(12)^. As a result, in 2008, Fulkerson and colleagues^(13)^ developed the Home Food Inventory (HFI) intending to create a more comprehensive inventory of foods and beverages in the home that could be completed by trained personnel. The HFI includes 190 items that measure thirteen major food categories, four ready access scores and one obesogenic score. The HFI was initially validated in school age children and adolescents (aged 10–17 years) and their parents using tests of construct validity. In short, they compared five HFI major food categories (e.g. dairy, vegetables (with and without potatoes), fruit, and meats and non-dairy protein) and the obesogenic score against a limited number of food and nutrient intake indicators (e.g. dairy and vegetable servings, energy, fibre, and vitamins A and C). All HFI scores examined were positively associated with parents’ food and nutrient intakes, while school age children and adolescents’ food and nutrient intakes were less consistently related to HFI scores (compared with parents); vegetable availability (with and without potatoes) were not associated with vitamin C or fibre, fruit availability was not associated with fruit servings, and meat and other non-dairy protein availability were not associated with non-dairy protein servings or protein.

The HFI is a comprehensive, useful tool for examining the physical elements of the home food environment, which was recently recognised by the American Society for Nutrition as an indicator of environmental influence on eating behaviours^(14)^. However, with the development of any measure, several limitations warrant attention. First, although the HFI scores were generally associated with food and nutrient intake, Fulkerson and colleagues^(13)^ only examined five out of thirteen major category scores from the HFI and compared the five scores against a limited number of food and nutrient intake indicators. Second, despite its use with early childhood samples^(15–19)^, the HFI was validated using a sample of 10–17-year-old children, who have different energy and nutrient needs than their younger counterparts. Relatedly, most of the early childhood research has largely relied on cross-sectional designs, limiting our understanding of changes in home food availability over time. Third, the obesogenic score from the HFI may need to be adjusted to account for dietary guidelines during early childhood. Early childhood is characterised by rapidly changing nutrient needs to support growth and development. As a result, recommendations for energy intake and intake of specific food groups differ throughout childhood^(10)^.

Limited research has examined home food availability using the HFI in samples focusing on early childhood^(15–19)^. Cross-sectional evidence revealed that increased availability of healthy foods was associated with healthy dietary patterns^(18)^ and consumption of fruits and vegetables^(19)^. Conversely, increased availability of unhealthy foods was associated with unhealthy dietary patterns^(18)^ and consumption of snack foods with high sugar and high fat^(19)^. Cepni and colleagues^(15)^ also found that healthy home food environments (including food availability) were positively associated with parental feeding practices (e.g. use of structure during feeding interactions). In contrast, obesogenic food availability was negatively associated with parental feeding practices. Two early childhood studies present feasibility and efficacy results from the FUNPALS Playgroup and the Prevention of Overweight in Infancy randomised control trials^(16,17)^; however, no significant differences or associations were found for home food availability. Although healthy and unhealthy food availability may indicate some aspects of a child’s diet, there is limited evidence that home food availability influences desired outcomes or improves because of an intervention. Previous research suggests two gaps in the literature that could be expanded. There is a lack of evidence examining whether home food availability changes during early childhood, and whether home food availability is associated with child food and nutrient intake during this time. There is some evidence of convergent and discriminant validity with early childhood samples, however, no studies have replicated the original analyses by Fulkerson and colleagues^(13)^.

The 2020–2025 Dietary Guidelines for Americans recommends that children should consume between 2 and 3 cups of whole milk per d (ages 12–24 months) or 2–2·50 cups of low-fat milk per d (ages 24–48 months)^(10)^. The HFI obesogenic score created by Fulkerson and colleagues^(13)^ includes all regular-fat dairy products (e.g. milk, yogurt and cheese). Although appropriate for older children, it may not be appropriate for children before or about 24 months of age. The evidence to consider dairy products as obesogenic foods or beverages is mixed. Clark and colleagues^(20)^ reviewed the literature on children ages 12–60 months. They found no association or an inverse association between milk consumption among preschool-aged children and subsequent overweight or obesity. Similarly, several studies reported evidence to suggest that milk consumption among children ages 3–10 years is linked to decreased risk for excess adiposity and obesity during adolescence^(21–23)^. Thus, it is important to consider whether the original scoring for an ‘obesogenic’ environment is appropriate for young children ages 24–48 months.

The objectives of the current study were twofold. Our first objective was to describe changes in home food availability during early childhood (ages 24–48 months), including using modified, developmentally sensitive obesogenic scores. Second, we sought to determine whether home food availability was associated with food servings and nutrient intakes of children concurrently at 24, 36 and 48 months. In the current study, we expand upon Fulkerson and colleagues^(13)^, initial findings by including a wider range of HFI scores, including the developmentally sensitive obesogenic scores, as well as food and nutrient intakes. We anticipated that the presence of energy-dense foods, including the obesogenic scores, will increase as children age, and these changes could influence their food and nutrient intake. In addition, we identified food servings and nutrients that should be correlated with the HFI scores of interest (Table 3 provides the full list). We anticipated that the HFI scores would positively correlate with the identified food servings and nutrient intakes, but we anticipated negative associations between energy-dense foods and fibre. An in-depth examination of the associations between the HFI scores and food and nutrient intakes was warranted to support using the HFI as an indicator of a young child’s diet.

## Methods

### Study design and sample

Data were drawn from a STRONG Kids 2 (SK2) longitudinal, birth cohort study on early childhood health and development; details about the SK2 programme can be found elsewhere^(24)^. Women were recruited from healthcare facilities and birthing classes during their third trimester of pregnancy from 2013 to 2017 in Central Illinois. Exclusion criteria included premature birth (< 37 weeks), birth conditions precluding normal feeding (e.g. cleft palate) and low birth weight (< 2·50 kg). Mothers provided written informed consent when registering for the study and were informed that they could withdraw anytime. Mothers were contacted via email or phone on follow-up dates, and a home visit was scheduled. The final sample includes 468 mothers and their infants starting from 1 week postpartum, and we utilised data from home visits and mother reports at 24, 36 and 48 months postpartum.

#### Home food inventory

Home food availability was assessed with the HFI^(13)^ at 24, 36 and 48 months; ‘home’ is defined as the dwelling where the parent(s) and child reside. The HFI is a structured checklist of food and beverage items available in the home; a trained research assistant completed this checklist with parents during a home visit. All items on the HFI were scored as ‘Yes’ (1 = item is available) and ‘No’ (0 = item is not available). For the current study objectives, we used fifteen categories/subscales: fruit, vegetables, vegetables excluding potatoes, dairy (regular fat; i.e. whole fat milk, cheese, yogurt, cream), dairy (reduced fat; i.e. reduced-fat milk (skim, 1 % and 2 % milk), cheese and yogurt), whole grains (bread and cereal), non-whole grains (bread and high sugar cereal), processed meats, other meats and non-dairy proteins, beverages (with sugar), candy, frozen desserts, prepared desserts, savoury snacks, and microwavable/quick-cook foods. We also used Fulkerson and colleagues’ original obesogenic score, which was calculated as the sum of regular-fat cheese, regular-fat milk, regular-fat yogurt, regular-fat other dairy, processed meat, regular-fat frozen desserts, regular-fat prepared desserts, high-sugar cereal, candy, and microwaveable/quick-cook food, as well as twenty-two individual items from added fats, savoury snacks, beverages, unhealthy kitchen accessibility, and unhealthy refrigerator accessibility. Because regular-fat dairy products are recommended for young children from 12 to 24 months^(10)^, we calculated and evaluated two alternative obesogenic scores at 24 months: (1) a version that excludes regular-fat milk and yogurt, and (2) a version that excludes both regular-fat milk, yogurt and cheese. Additional information regarding scoring of the HFI can be found elsewhere^(13)^.

#### Block FFQ

Child consumption of foods and nutrient intake was assessed with the Nutrition Quest Child Block FFQ for ages 2–7 years^(25)^ at 24, 36 and 48 months. The FFQ includes ninety items to ascertain information related to the child’s ‘usual eating habits in the past 6 months’ using a 1 (never) to 8 (every day) scale; mothers completed an online version of the FFQ. The National Health and Nutrition Examination Survey (NHANES) III dietary recall data were used by Nutrition Quest to estimate food servings per d, and the USDA Nutrient Database for Standard Reference was used for nutrient assessment. All food serving items were coded as how often that item is eaten per week, and both unadjusted and energy-adjusted estimates of micro- and macronutrient intakes were included. For the current study objectives, we used twenty-seven food servings and eighteen micro- and macronutrients. Example servings include fruits, vegetables, dairy (milk), other dairy (cheese and ice cream), whole grains (oz.), any grains (oz.), Lunchables®, hot dog or sausage, red meat, poultry, sugar-sweetened beverages, chocolate candy, ice cream, cookies and savoury snacks. Examples of micro- and macronutrients include vitamins A, C, and D, Ca, K, Na, Fe, fibre, protein, saturated fat, added sugars, total energy (kilocalories [kcal]), and percent kcal from protein, saturated fat, fat, and sweets.

### Analysis plan

First, descriptive statistics and then changes in the HFI over time were examined using repeated-measures ANOVA (RMANOVA) from 24 to 48 months for the scores of interest: availability of fruit, vegetables, vegetables excluding potatoes, dairy, dairy (reduced fat), whole grains (bread and cereal), non-whole grains (bread and high-sugar cereal), processed meats, other meats and non-dairy proteins, beverages (with sugar), candy, frozen desserts, prepared desserts, savoury snacks, and microwavable/quick-cook foods, obesogenic score (v1), obesogenic score (v2), and obesogenic score (v3). A Bonferroni correction was applied to account for multiple *post hoc* pairwise comparisons.

Second, following the analyses by Fulkerson and colleagues (2008), Spearman’s correlations were used to examine the associations between the HFI scores of interest (e.g. fruit, vegetables, dairy, whole grains, processed meats, microwaveable/quick cook foods and three obesogenic scores (two modified versions)) and the FFQ food servings and nutrient intakes (e.g. vitamin C, Ca, K, protein and saturated fat) from the FFQ; the Spearman correlations were conducted separately at 24, 36 and 48 months. A more comprehensive list of both HFI scores and FFQ food servings and nutrients were selected to expand upon the initial validation of the HFI and to provide evidence of construct validity for using the HFI in households with young children. Again, to account for multiple pairwise comparisons, a Bonferroni correction was applied; the unadjusted and adjusted estimates are provided and discussed.

Data management and data analysis were conducted using Stata 17^27^. Given the longitudinal nature of the data, there is some missing data; over time, missing data for the HFI and FFQ ranged from 12 % to 21 % and 28 % to 38 %, respectively. Missing data are likely due to attrition at the later time points of data collection. The FFQ was also an additional questionnaire that mothers completed after their regular annual surveys, which may have resulted in a slightly lower response rate. Missing data analyses were conducted to determine whether any sociodemographic variables (i.e. child sex, monthly household income, and maternal perceived social status, employment status and age at 6 weeks) should be accounted for in the RMANOVAs; demographics with reasonable variability were selected to examine differences. Monthly household income and maternal employment status and age at 6 weeks were significantly associated with missingness in the primary variables (HFI). The RMANOVAs were conducted with and without the covariates, and although the findings did not meaningfully differ, the listwise deletion procedure resulted in a substantial reduction in observations (*k* = 1174 without covariates compared with *k* = 811 with covariates) (see online supplementary material, Supplemental Table 1 for adjusted *F*-tests). Considering the descriptive, rather than predictive, nature of the study, the RMANOVAs without covariates will be presented.

## Results

### Sample characteristics

Table 1 provides descriptive statistics for child and maternal characteristics.

**Table 1:** Child and maternal characteristics reported at 6 weeks postpartum (*n* 468)

	*n*	%
Child characteristics		
Child sex: male	239	51 %
Exclusively breastfed for 6 weeks	317	68 %
Maternal characteristics		
Race		
Alaska Native, American Indian or Native Hawaiian	8	2 %
Asian or Asian American	39	8 %
Black or African American	27	6 %
White	375	80 %
Unknown	19	4 %
Education		
Bachelor’s or graduate degree	341	73 %
Some college/technical school	88	19 %
Grade school/high school	32	7 %
Unknown	7	2 %
Marital status		
Not single	414	89 %
Single	45	10 %
Unknown	9	2 %
WIC participation (mother, child or both)	97	21 %
Monthly household income		
≤ $3000	137	29 %
$3000–$5000	123	26 %
≥ $5001	155	33 %
Unknown	53	11 %
Maternal pre-pregnancy BMI		
Not having overweight or obesity	217	46 %
Having overweight	175	37 %
Having obesity	198	42 %
Unknown	20	4 %

WIC, Special Supplemental Nutrition Program for Women, Infants, and Children.

Percentages may exceed 100 % due to rounding. Unknown data were not provided by the mother. Maternal BMI (in kg/m^2^) was classified as not having overweight or obesity (underweight [BMI < 18·5] and ‘normal’ weight [18·5 ≤ BMI < 25]), having overweight (25 ≤ BMI < 30) and having obesity (BMI ≥ 30).

### Change in home food availability from 24 to 48 months

**Fig. 1:**
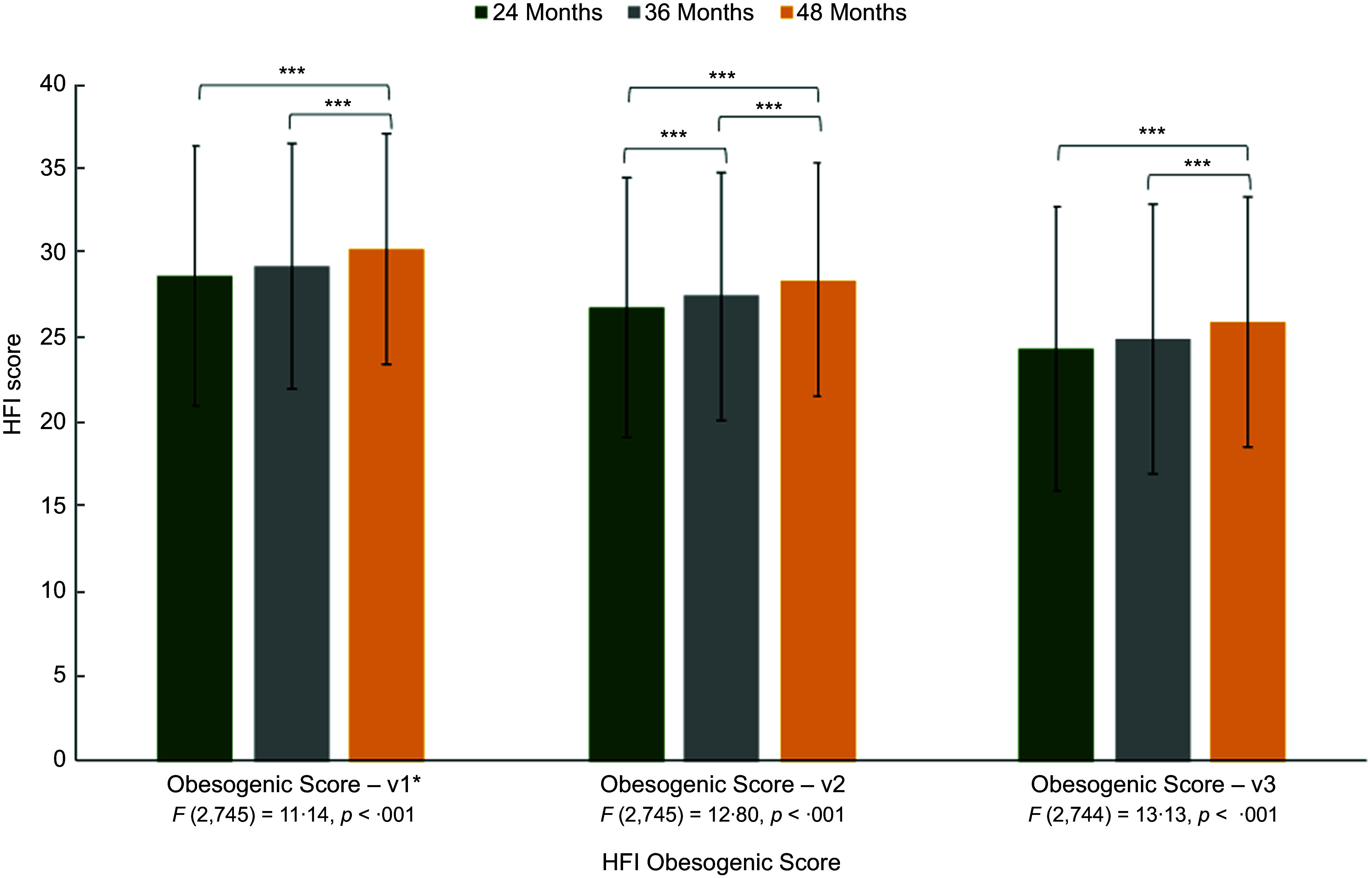
Changes in the Home Food Inventory (HFI) obesogenic scores across from 24 to 48 months. Results of the repeated-measures ANOVAs are provided under the x-axis. *Post hoc* pairwise comparisons with a Bonferroni correction were used and presented above the bars in the figure.***P < 0·001

Table 2 and Fig. 1 present the changes in the HFI groups and obesogenic scores, respectively, at 24, 36 and 48 months. Seven of the HFI scores demonstrated significant changes over time. The presence of vegetables, both with and without potatoes, was significantly higher at 48 months compared with 24 and 36 months, as were non-whole grains (bread and high-sugar cereal), processed meats and savoury snacks. Candy and microwavable/quick-cook foods were also more common in the home at 36 and 48 months compared with 24 months. We present the full results of the RMANOVA with pairwise comparison tests (including *F*-tests and *t*-test *P*-values) in Table 2.

**Table 2:** Descriptive statistics and results from repeated-measures ANOVAs for HFI category scores at 24, 36 and 48 months

HFI category		24 months (*n* 413)		36 months (*n* 389)		48 months (*n* 370)		Change over time	
*Total number of (items) in home*	Range	*M*	sd	*M*	s d	*M*	s d	*F*	*P*-value
Fruits	0–26	9·62	3·80	9·91	3·90	10·05	3·96	*F* (2, 743) = 1·57	0·209
Vegetables	0–20	10·41*	3·48	10·63*	3·54	10·97^†^	3·59	*F* (2, 741) = 3·93	**0·020**
Vegetables – excl. potatoes	0–9	9·64*	3·35	9·90*	3·38	10·22^†^	3·44	*F* (2, 741) = 4·25	**0·015**
Dairy – regular fat	0–9	4·31	1·67	4·34	1·75	4·41	1·73	*F* (2, 745) = 0·47	0·627
Dairy – reduced fat	0–12	3·13	1·88	3·17	1·74	3·05	1·87	*F* (2, 745) = 0·72	0·489
Whole grains – bread, WW cereal	0–6	2·00	1·27	1·93	1·19	1·97	1·20	*F* (2, 742) = 0·83	0·438
Non-whole grains – bread, HS cereal	0–8	3·74*	1·58	3·87*	1·65	4·11^†^	1·62	*F* (2, 743) = 8·32	**<0·001**
Processed meats	0–4	1·60*	1·06	1·66*	1·12	1·77^†^	1·08	*F* (2, 740) = 4·49	**0·012**
Other meats and non-dairy proteins	0–12	6·78	1·60	6·94	1·69	6·86	1·59	*F* (2, 743) = 1·15	0·319
Beverages – regular sugar	0–6	2·03	1·24	1·99	1·20	2·02	1·28	*F* (2, 743) = 0·35	0·706
Candy	0–5	2·09*	1·56	2·47^†^	1·59	2·60^†^,^‡^	1·62	*F* (2, 741) = 27·45	**<0·001**
Frozen desserts	0–3	0·78	0·69	0·80	0·62	0·89	0·64	*F* (2, 741) = 2·97	0·052
Prepared desserts	0–6	1·26	1·04	1·33	1·06	1·28	1·04	*F* (2, 743) = 0·48	0·618
Savoury snacks	0–10	4·93*	1·87	5·08*	1·93	5·34^†^	1·90	*F* (2, 743) = 8·14	**<0·001**
Microwavable/quic*k*-cook foods	0–9	2·21*	1·67	2·44^†^	1·72	2·66^†^,^‡^	1·80	*F* (2, 741) = 16·62	**<0·001**

HFI, Home Food Inventory; WW, whole wheat; HS, high sugar.

*,†,‡Within a row, means without a common superscript differ at *P* < 0·05.

Bolded values are significant at *P* < 0·05 or less.

All HFI scores are calculated as sum scores based on the original instructions by Fulkerson and colleagues (2008). Frozen desserts, prepared desserts and savoury snacks only include ‘regular-fat’ items; ‘reduced-fat’ items were not included in those three scores. Two modifications were made for whole grains and non-whole grains where whole wheat cereal and high sugar cereals were added to their respective categories.

*Post hoc* pairwise comparisons with a Bonferroni correction were used.

The original obesogenic score (v1)*, with all regular-fat dairy products, significantly increased from 24 to 36 and 48 months. The modified obesogenic scores revealed similar patterns. Compared with the original obesogenic score (v1), the mean scores decrease (with items removed) with regular-fat milk and yogurt removed (v2) and with regular-fat milk, yogurt, and cheese removed (v3). However, the linear trend is consistent such that the obesogenic scores are lowest at 24 months compared with 36 and 48 months. The full results of the RMANOVA with pairwise comparison tests (including *F*-tests and *t*-test *P*-values) are demonstrated in Fig. 1.

### Associations between home food availability and child food and nutrient intake

Results of the Spearman correlations are presented in Table 3, including both unadjusted and Bonferroni-adjusted estimates.

**Table 3:** Spearman’s correlations between HFI category scores and FFQ servings per week and nutrients at 24, 36 and 48 months

		24 months		36 months		48 months	
HFI category	FFQ servings and nutrients	Unadjusted *ρ*	Adjusted *ρ*	Unadjusted *ρ*	Adjusted *ρ*	Unadjusted *ρ*	Adjusted *ρ*
Fruits	Fruit servings*	**0·28**	**0·28**	**0·24**	**0·24**	**0·30**	**0·30**
	Vitamin A	**0·15**	0·15	**0·16**	0·16	**0·22**	**0·22**
	Vitamin C	**0·17**	**0·17**	0·03	0·03	**0·12**	0·12
	Fibre from fruit and veg	**0·29**	**0·29**	**0·21**	**0·21**	**0·24**	**0·24**
	Potassium	0·08	0·08	0·02	0·02	0·01	0·01
Vegetables	Vegetable servings	**0·33**	**0·33**	**0·36**	**0·36**	**0·34**	**0·34**
	Vitamin A	**0·26**	**0·26**	**0·18**	**0·18**	**0·38**	**0·38**
	Vitamin C	0·07	0·07	0·01	0·01	**0·14**	0·14
	Fibre from fruit and veg	**0·24**	**0·24**	**0·22**	**0·22**	**0·36**	**0·36**
	Potassium	0·09	0·09	−0·05	−0·05	**0·15**	0·15
Vegetables – excl. potatoes	Vegetable servings	**0·33**	**0·33**	**0·36**	**0·36**	**0·34**	**0·34**
	Vitamin A	**0·26**	**0·26**	**0·17**	**0·17**	**0·38**	**0·38**
	Vitamin C	0·07	0·07	0·02	0·02	0·14	0·14
	Fibre from fruit and veg	**0·25**	**0·25**	**0·23**	**0·23**	**0·36**	**0·36**
	Potassium	0·09	0·09	−0·05	−0·05	**0·16**	0·16
Dairy – regular fat	Dairy servings	0·05	0·05	−0·02	−0·02	0·10	0·10
	Other dairy servings	0·10	0·10	**0·12**	0·12	**0·20**	**0·20**
	Vitamin D	0·03	0·03	0·03	0·03	−0·03	−0·03
	Ca	0·02	0·02	−0·04	−0·04	−0·01	−0·01
	Protein (NA)	0·10	0·10	0·07	0·07	0·13	0·13
	Potassium	−0·09	−0·09	**−0·12**	−0·12	**−0·16**	−0·16
	Saturated fat (NA)	**0·19**	**0·19**	**0·17**	0·17	**0·21**	**0·21**
	kcal from pro. (% total kcal)	−0·01	−0·01	**−0·11**	−0·11	−0·03	−0·03
	kcal from sat fat (% total kcal)	**0·18**	**0·18**	**0·19**	**0·19**	**0·21**	**0·21**
Dairy – reduced fat	Dairy servings	0·11	0·11	0·09	0·09	**0·19**	**0·19**
	Other dairy servings	**0·17**	0·17	**0·15**	0·15	**0·15**	0·15
	Vitamin D	−0·01	−0·01	−0·03	−0·03	0·004	0·004
	Ca	0·09	0·09	**0·24**	**0·24**	**0·19**	**0·19**
	Potassium	**−0·12**	−0·12	0·01	0·01	0·06	0·06
	Protein (NA)	−0·03	−0·03	0·03	0·03	0·07	0·07
	kcal from pro. (% total kcal)	0·08	0·08	**0·21**	**0·21**	**0·18**	0·18
Whole grains – bread and cereal	Total whole grains (oz.)	0·11	0·11	**0·20**	**0·20**	**0·16**	0·16
	Total any grains (oz.)	**0·11**	0·11	**0·13**	0·13	0·08	0·08
	Sweet cereal servings	0·01	0·01	0·09	0·09	−0·03	−0·03
	Cold cereal servings	**0·20**	**0·20**	**0·23**	**0·23**	**0·25**	**0·25**
	Total fibre	0·07	0·07	0·06	0·06	**0·22**	**0·22**
	Fibre from grains	0·11	0·11	0·11	0·11	0·05	0·05
	Added sugar	0·03	0·03	0·09	0·09	−0·08	−0·08
Non-whole grains – bread and high-sugar cereal	Total non-whole grains (oz.)	0·10	0·10	**0·15**	0·15	0·07	0·07
	Total any grains (oz.)	0·09	0·09	**0·15**	0·15	0·07	0·07
	Sweet cereal servings	**0·25**	**0·25**	**0·33**	**0·33**	**0·28**	**0·28**
	Cold cereal servings	**0·17**	0·17	**0·13**	0·13	0·11	0·11
	Total fibre	**−0·14**	−0·14	**−0·11**	−0·11	−0·09	−0·09
	Fibre from grains	0·05	0·05	0·11	0·11	−0·06	−0·06
	Added sugar	0·11	0·11	**0·19**	**0·19**	**0·21**	**0·21**
	kcal from sweets (% total kcal)	0·10	0·10	**0·15**	0·15	**0·12**	0·12
Processed meats	Lunchmeat servings	**0·20**	0·20	**0·14**	0·14	**0·22**	0·22
	Lunchables® servings	**0·14**	0·14	**0·16**	0·16	**0·23**	0·23
	Bacon/breakfast saus. servings	**0·42**	**0·42**	**0·34**	**0·34**	**0·33**	**0·33**
	Hot dog/sausage servings	**0·48**	**0·48**	**0·31**	**0·31**	**0·42**	**0·42**
	Protein (NA)	0·07	0·07	0·03	0·03	0·05	0·05
	Saturated fat (NA)	**0·14**	0·14	0·09	0·09	**0·12**	0·12
	Na	0·06	0·06	0·06	0·06	0·02	0·02
	kcal from pro. (% total kcal)	0·03	0·03	0·03	0·03	0·05	0·05
	kcal from sat fat (% total kcal)	**0·15**	0·15	**0·19**	**0·19**	**0·21**	0·21
	kcal from fat (% total kcal)	**0·17**	0·17	**0·18**	0·18	**0·15**	0·15
Other meats and non-dairy protein	Red meat servings	0·10	0·10	0·07	0·07	0·10	0·10
	Poultry servings	0·11	0·11	0·03	0·03	**0·13**	0·13
	Fish servings	**0·24**	**0·24**	**0·30**	**0·30**	**0·26**	**0·26**
	Eggs servings	0·07	0·07	0·06	0·06	**0·12**	0·12
	Legumes servings	0·10	0·10	**0·12**	0·12	0·07	0·07
	Protein (NA)	0·09	0·09	**0·13**	0·13	0·05	0·05
	Vitamin D	−0·07	−0·07	−0·03	−0·03	−0·03	−0·03
	Fe	0·02	0·02	0·05	0·05	−0·01	−0·01
	kcal from pro. (% total kcal)	0·09	0·09	0·01	0·01	**0·18**	0·18
Beverages – incl. sugary	SSB servings	**0·31**	**0·31**	**0·36**	**0·36**	**0·29**	**0·29**
	SSB per d (g)	**0·31**	**0·31**	**0·36**	**0·36**	**0·30**	**0·30**
	SSB per d (cals)	**0·31**	**0·31**	**0·36**	**0·36**	**0·29**	**0·29**
	Added sugars	**0·11**	0·11	0·11	0·11	**0·13**	0·13
	kcal from sweets (% total kcal)	**0·15**	0·15	**0·14**	0·14	**0·20**	**0·20**
Candy	Chocolate candy servings	**0·29**	**0·29**	**0·26**	**0·26**	**0·32**	**0·32**
	Non-chocolate candy servings	**0·35**	**0·35**	**0·40**	**0·40**	**0·37**	**0·37**
	Saturated fat (NA)	−0·03	0·05	**0·11**	0·16	0·03	0·03
	Added sugars	**0·11**	0·11	**0·19**	**0·19**	**0·27**	**0·27**
	kcal from sat fat (% total kcal)	−0·03	−0·03	**0·11**	0·11	0·03	0·03
	kcal from fat (% total kcal)	−0·03	−0·03	0·07	0·07	−0·06	−0·06
	kcal from sweets (% total kcal)	**0·15**	**0·15**	**0·23**	**0·23**	**0·26**	**0·26**
Frozen desserts	Ice cream servings	**0·17**	0·17	**0·19**	**0·19**	**0·18**	0·18
	Popsicle servings	**0·12**	0·12	0·09	0·09	0·11	0·11
	Saturated fat (NA)	**0·15**	0·15	**0·20**	**0·20**	**0·14**	0·14
	Added sugars	**0·15**	0·15	0·01	0·01	**0·12**	0·12
	kcal from sat fat (% total kcal)	0·02	0·02	0·11	0·11	**0·14**	0·14
	kcal from fat (% total kcal)	−0·01	−0·01	0·08	0·08	0·11	0·11
	kcal from sweets (% total kcal)	**0·11**	0·11	0·05	0·05	0·11	0·11
Prepared desserts	Cookies per d (g)	**0·25**	**0·25**	**0·18**	**0·18**	**0·30**	**0·30**
	Donuts and cake servings	**0·21**	**0·21**	**0·25**	**0·25**	**0·32**	**0·32**
	Saturated fat	0·01	0·02	0·08	0·12	−0·02	0·12
	Added sugars	**0·17**	0·17	**0·11**	0·11	**0·23**	**0·23**
	kcal from sat fat (% total kcal)	0·01	0·01	0·08	0·08	−0·02	−0·02
	kcal from fat (% total kcal)	−0·02	−0·02	0·07	0·07	0·004	0·004
	kcal from sweets (% total kcal)	**0·20**	**0·20**	**0·15**	0·15	**0·31**	**0·31**
Savoury snacks	Savoury snack servings	**0·29**	**0·29**	**0·28**	**0·28**	**0·34**	**0·34**
	Saturated fat (NA)	0·06	0·06	0·11	0·11	**0·13**	0·13
	Added sugars	**0·11**	0·11	**0·16**	0·16	**0·16**	0·16
	Na	0·05	0·05	0·04	0·04	0·03	0·03
	kcal from sat fat (% total kcal)	0·02	0·02	−0·001	−0·001	−0·004	−0·004
	kcal from fat (% total kcal)	0·04	0·04	−0·03	−0·03	0·01	0·01
	kcal from sweets (% total kcal)	**0·13**	0·13	**0·14**	0·14	**0·14**	0·14
Microwavable/quick-cook foods	Saturated fat (NA)	0·03	0·03	**0·17**	0·17	**0·13**	0·13
	Added sugars	**0·16**	0·16	**0·22**	**0·22**	**0·23**	**0·23**
	Na	−0·02	−0·02	−0·03	−0·03	−0·03	−0·03
	Total fibre	**−0·30**	**−0·30**	**−0·24**	**−0·24**	**−0·31**	**−0·31**
	kcal from sat fat (% total kcal)	−0·05	−0·05	0·01	0·01	0·05	0·05
	kcal from fat (% total kcal)	−0·07	−0·07	−0·04	−0·04	0·02	0·02
	kcal from sweets (% total kcal)	**0·25**	**0·25**	**0·20**	**0·20**	**0·24**	**0·24**
Obesogenic score – version 1 (incl. all regular fat dairy)	Energy (kcal) (NA)	**0·18**	**0·18**	**0·19**	**0·19**	**0·25**	**0·25**
	Saturated fat (NA)	**0·16**	0·16	**0·23**	**0·23**	**0·25**	**0·25**
	Added sugars	**0·16**	0·16	**0·22**	**0·22**	**0·31**	**0·31**
	Na	−0·01	−0·01	−0·01	−0·01	−0·04	−0·04
	kcal from sat fat (% total kcal)	0·07	0·07	**0·17**	0·17	**0·13**	0·13
	kcal from fat (% total kcal)	0·04	0·04	**0·13**	0·13	0·06	0·06
	kcal from sweets (% total kcal)	**0·21**	**0·21**	**0·25**	**0·25**	**0·32**	**0·32**
Obesogenic score – version 2 (excl. milk/dairy beverages and yogurt)	Energy (kcal) (NA)	**0·16**	0·16	**0·17**	0·17	**0·25**	**0·25**
	Saturated fat (NA)	**0·14**	0·14	**0·21**	**0·21**	**0·24**	**0·24**
	Added sugars	**0·18**	**0·18**	**0·22**	**0·22**	**0·32**	**0·32**
	Na	0·001	0·001	0·01	0·01	−0·05	−0·05
	kcal from sat fat (% total kcal)	0·04	0·04	**0·15**	0·15	0·10	0·10
	kcal from fat (% total kcal)	0·01	0·01	0·11	0·11	0·03	0·03
	kcal from sweets (% total kcal)	**0·23**	**0·23**	**0·26**	**0·26**	**0·33**	**0·33**
Obesogenic Score – version 3 (excl. milk/dairy beverages, yogurt, and cheese)	Energy (kcal) (NA)	**0·16**	0·16	**0·18**	**0·18**	**0·25**	**0·25**
	Saturated fat (NA)	**0·13**	0·13	**0·21**	**0·21**	**0·23**	**0·23**
	Added sugars	**0·19**	**0·19**	**0·24**	**0·24**	**0·30**	**0·30**
	Na	0·01	0·01	0·02	0·02	−0·05	−0·05
	kcal from sat fat (% total kcal)	0·03	0·03	**0·14**	0·14	0·10	0·10
	kcal from fat (% total kcal)	0·02	0·02	0·10	0·10	0·04	0·04
	kcal from sweets (% total kcal)	**0·24**	**0·24**	**0·27**	**0·27**	**0·32**	**0·32**

HFI, Home Food Inventory; NA, not energy-adjusted; oz., ounce equivalent; SSB, sugar-sweetened beverages; cals, calories; Pro, protein.

* FFQ food servings are coded as per week unless otherwise denoted as ounces (ounce equivalent), grams, calories or kcal. All FFQ nutrients have been energy-adjusted unless otherwise denoted as NA.

Bolded estimates indicate significant correlations at *P* < 0·05 or less.

Unadjusted *ρ*, Spearman’s correlations without Bonferroni adjustment; adjusted *ρ,* Bonferroni adjustment applied to Spearman’s correlations.

Savoury snack servings = Lunchables®, snacks like potato chips, corn chips, popcorn and pretzels.

HFI sample sizes were 413, 389 and 370 at 24, 36 and 48 months, respectively; FFQ sample sizes were 337, 317 and 289 at 24, 36 and 48 months, respectively.

#### Fruits and vegetables

The HFI fruit and vegetable scores (with and without potatoes) were positively associated with fruit and vegetable servings, vitamin A, and fibre from fruits and vegetables over time (both unadjusted and adjusted). Vitamin C was associated with fruit availability at 24 months and with fruit and vegetable (with potatoes) availability at 48 months (unadjusted). Both vegetable scores were associated with potassium at 48 months (unadjusted).

#### Dairy

Only the HFI reduced-fat dairy score was associated with dairy servings at 48 months, but both regular-fat and reduced-fat dairy availability were associated with other dairy servings at 36 and 48 months; the correlations between regular-fat dairy and other dairy servings remained significant even after adjustment. Regular-fat dairy availability was consistently associated with saturated fat and percent kcal from saturated fat. In contrast, only reduced-fat dairy was associated with Ca at 36 and 48 months and with percent kcal from protein at 36 months. Regular-fat and reduced-fat dairy scores demonstrated some negative associations with potassium at 36 and 48 months, and at 24 months, respectively (unadjusted).

#### Grains

The HFI whole grains score demonstrated associations with whole grain intake (ounces) at 36 and 48 months (only 36 months was significant after adjustment) and with any grains (ounces) at 24 and 36 months (unadjusted). Whole grain availability was consistently associated with cold cereal servings over time, and conversely, non-whole grains were associated with sweet cereal servings. Whole grains were also linked to higher fibre intake at 48 months, while non-whole grains were linked to lower fibre intake at 24 and 36 months (unadjusted). Non-whole grain availability, which included high-sugar cereals, was associated with added sugars at 36 and 48 months, as well as the percent kcal from sweets at 36 months (unadjusted).

#### Processed meats and other meats and non-dairy proteins

HFI processed meat scores were consistently associated with bacon or breakfast sausage and hot dog or sausage servings. At the same time, the associations with lunchmeat and Lunchables® were only significant when unadjusted. The availability of processed meats was associated with percent kcal from saturated fat and regular fat, however, mostly when unadjusted. Other meats and non-dairy proteins from the HFI did not demonstrate many associations with servings and nutrient intakes, except for fish servings over time; unadjusted associations include poultry and egg servings and percent kcal from protein at 48 months and legume servings and protein at 36 months.

#### Sugar-sweetened beverages and foods

Both the HFI beverage (including sugary beverages) and candy scores appeared to be an indicator of servings over time: beverage availability was linked to servings and consumption of sugar-sweetened beverages per d in both grams and calories. However, the association between beverages and added sugars was smaller in magnitude and not consistently significant at 24 and 36 months. Still, by 48 months, beverage availability was strongly associated with the percent kcal from sweets compared with the earlier time points. Unlike beverages, candy availability was consistently linked to added sugars and percent kcal from sweets.

The HFI prepared dessert score demonstrated stronger and more significant associations with servings and nutrients than the frozen dessert score. The availability of prepared desserts was associated with grams of cookies consumed per d and with donut and cake servings, over time. Frozen desserts were most linked to ice cream servings rather than popsicle servings and saturated fat (unadjusted). Prepared desserts were also associated with added sugars (mostly unadjusted) and with percent kcal from sweets, but frozen desserts demonstrated smaller and mostly non-significant associations with added sugars and percent kcal from sweets.

#### Savoury snacks and microwavable foods

The HFI savoury snack score appeared to be an indicator of savoury snack servings. The availability of savoury snacks and microwavable/quick-cook foods were associated with added sugars and percent kcal from sweets (unadjusted only for savoury snacks). Consistently, microwavable/quick-cook foods were negatively related to fibre intake.

#### Obesogenic scores

All three versions of the obesogenic scores were associated with energy (kcal), saturated fat and added sugars at 24 months before adjustment. After adjustment, the original obesogenic score (v1) remained significant for energy, and the modified obesogenic scores (v2 and v3) remained significant for added sugars. At 36 and 48 months, all versions of the obesogenic scores were associated with energy, saturated fat and added sugars. The positive association between the obesogenic scores and percent kcal from sweets was consistent and positive over time, while associations between the obesogenic scores and percent kcal from saturated fat or regular fat were inconsistent.

## Discussion

A recent report from the American Society for Nutrition underscores the importance of diversity in research methods used to examine nutrition^(14)^. The HFI^(13)^ has been used as a gauge of environmental influence on eating behaviours and with samples of children, adolescents, and adults. Yet, there is limited research focusing on early childhood. The existing research heavily relies on cross-sectional designs and broad scores of the HFI (healthy *v*. unhealthy foods) and child food and nutrient intake. To our knowledge, this is the first study to examine changes in home food availability from 24 to 48 months of age and associations between home food availability and child food and nutrient intake at 24–48 months. Results indicate that the availability of less nutritious foods increases as children age, which was apparent in both the individual food group scores and the obesogenic scores, and that the availability of both nutritious and less nutritious foods were associated with child food and nutrient intake.

The changes identified in home food availability suggest that parents may have increased access to and/or purchase less nutritious foods as children age. Food items such as non-whole grains, processed meats, savoury snacks, candy and microwavable/quick-cook foods were more commonly available in the home at 48 months compared with 24 and/or 36 months. The availability of energy-dense or sugary foods may continue to rise from early childhood to adolescence; in the USA, children (ages 2–6 years) and adolescents have demonstrated increased consumption of energy-dense foods across several decades^(26,27)^. The presence of these items contributed to higher obesogenic scores over time, with the most significant changes demonstrated for the modified obesogenic score (v2 – excluding milk and yogurt from the score). The obesogenic scores observed in the current study are similar to those reported for early childhood^(17)^ and school-age children^(28)^; others have used the obesogenic score but did not report descriptive statistics (i.e. means and standard deviations for the obesogenic score). It is also worth noting that the availability of vegetables increased as children aged. This evidence is promising as a potential strategy to offset the availability and consumption of less nutritious foods that are also increasing.

Overall, the HFI food group scores were associated with food servings from the FFQ in the hypothesised direction, but the associations between the HFI and nutrient intake were less consistent. In particular, the availability of regular-fat dairy and other meats and non-dairy protein do not translate to the hypothesised food and nutrient intakes for children in this sample. Unlike Fulkerson and colleagues^(13)^ findings, regular-fat dairy was only (and rarely) associated with ‘other’ dairy servings (i.e. cheese and ice cream) and was not associated with vitamin D or Ca. The lack of associations found with regular-fat dairy, other meats, and non-dairy protein, and non-whole grains may be due to a mismatch between availability and intake (or intake estimation). Most children ages 24–48 months do not meet the recommended intake for dairy but do meet the recommended intake for protein, while their intake of grains is driven by refined grains^(10)^. Two possible reasons for these discrepancies could be due to measurement. The presence of foods in the home may be more reflective of the parents’ diet, rather than the child’s diet. Since, adults tend to consume more non-whole grains^(10,29)^, they may be more likely to stock their homes with non-whole grains. In addition, the Block FFQ relies on parent reports of how often their child consumes a particular food item, and then Nutrition Quest estimates servings and nutrient intake from parent reports. Thus, it is hard to know precisely how often parents offer foods to their child or how often the child consumes foods, but it may be unlikely that parents offer dairy, protein (not processed) and refined grains to their children daily^(30)^.

Availability of fruits and vegetables (with and without potatoes) was consistently associated with food and nutrient intake, which is promising as children are increasingly exposed to vegetables as they age. The observed associations between fruit and vegetable scores with food servings and intake of vitamins A and/or C and fibre from fruits and vegetables offer evidence of construct validity for the HFI fruit and vegetable scores for use with young children. Fulkerson and colleagues^(13)^ observed similar associations, but the availability of vegetables in the home was not associated with adolescent fibre intake. This finding is similar to other studies demonstrating that the availability of nutritious foods, such as fruits and vegetables, is associated with greater fruit and vegetable consumption^(19,31–33)^ and overall diet quality^(19)^ in samples of children ranging from 2 to 16 years-of-age.

Unlike previous studies, we provided a more thorough examination of both HFI scores and indicators of food and nutrient intake. By not relying on broad scores of less nutritious foods, we demonstrated that the availability of beverages with sugar, candy, prepared desserts, savoury snacks and microwavable/quick-cook foods were consistent indicators of food servings and the intake of added sugars and kcal from sweets. Similarly, the availability of processed meats was strongly indicative of bacon, sausage, and/or hot dog servings but were also associated with lunchmeat and Lunchables® servings and kcal from saturated fat and fat. Others have reported similar associations between the availability of less nutritious foods and consuming foods or beverages high in sugar or fat among children ages 2–10 years^(18,19,34)^. Increased availability of less nutritious foods, such as processed meats, savoury snacks, candy and microwavable/quick-cook foods, may lead to decreased quality of diets in young children.

A sub-aim of our second objective was to examine the usefulness of modified obesogenic scores. The obesogenic score created by Fulkerson and colleagues^(13)^ included all regular-fat dairy products, but as previously mentioned, this approach may not be the most developmentally sensitive scoring for younger children^(10)^. Two modified versions of the obesogenic scores were created and examined to determine whether the omission of regular-fat dairy items would produce nuanced findings. However, all three versions of the obesogenic scores were related to energy intake, saturated fat, added sugars and kcal from sweets over time. The magnitude of the correlation between the obesogenic score and energy intake is stronger, yet complementary to the original findings by Fulkerson and colleagues^(13)^. Our finding that higher obesogenic scores were indicative of saturated fat and added sugar intake is in line with past research focusing on high-fat snack intake (using behavioural observations)^(35)^ and decreased diet quality (using dietary recalls)^(36)^ among children. Because the modified obesogenic scores were not distinguishable from the original score, we recommend that researchers only use the modified scores for children ages 12–24 months, when regular-fat dairy is recommended. Researchers may choose to use the modified obesogenic scores if interested in the nuances of dairy availability and consumption.

Several factors that may influence or interact with home food availability should be considered in future research^(2)^. Child and parent characteristics, such as picky eating or feeding practices, can alter home food availability and the child’s diet. For example, children who are characterised as ‘picky eaters’ may request that parents purchase and offer foods that they consider to be more palatable^(37–39)^. Early childhood is a critical time when children develop their food preferences and dietary patterns. During this time, parents are tasked with the responsibility of choosing which foods to purchase and offer to their children^(6)^ as well as deciding how to offer those foods via their feeding practices^(40)^. For example, the use of parental control during feeding (e.g. restricting food) has been linked to increased intake of less nutritious foods and risk for dysregulated eating^(41)^ and overweight and obesity^(42–44)^. External characteristics, such as stress, economic hardship, food insecurity and food accessibility, may alter home food availability. Jang and colleagues^(18)^ found that parental stress was negatively associated with the availability of healthy foods, such as fruit, vegetables, and healthy snacks and beverages. Children whose families experience economic hardships, food insecurity, and/or have reduced neighbourhood access to fruits and vegetables may have decreased home availability of nutritious foods and be at greater risk for overweight and obesity^(45,46)^. Although not directly relevant to the current study sample, Agarwal and colleagues^(46)^ stress the importance of considering the neighbourhood food environments as it relates to the home food environment. They reported that limited access to fresh fruits and vegetables was associated with reduced availability of nutritious foods at home, decreased diet quality among children, and higher child BMI, and that these associations were strengthened for children who experience household food insecurity. Among families who experience food insecurity, participation in SNAP may be beneficial for improving parents’ purchasing behaviours, home availability of nutritious foods and beverages, and child diet^(47)^.

## Strengths and limitations

Strengths of the current study include having access to a longitudinal, birth cohort study of children and their families, with 62 %–79 % of the birth cohort providing HFI and FFQ data during early childhood. The study design, sample size and measures collected allowed for a more robust examination of home food availability and child intake over time. In addition, the Bonferroni-adjusted Spearman’s correlations allowed us to determine which HFI and FFQ estimates were consistently correlated with one another, while accounting for multiple pairwise comparisons. The findings from this study underscore the importance of understanding whether the physical home food environment influences child diet over time and this may have implications for later child health outcomes.

Several limitations must be acknowledged. First, the demographic characteristics of the SK2 birth cohort lack variability in race/ethnicity, education, marital status and household income. Mothers in the SK2 sample who were younger, not currently employed full time, and had a lower monthly household income demonstrated greater attrition over time compared with their counterparts. As such, the findings presented in the current study are not generalisable to families who have younger mothers and may experience greater economic hardships. Future efforts are needed to improve the diversity in longitudinal, birth cohort studies, and to generalise to a greater range of children and families. Second, the correlation analysis limits what inferences can be made about the association between home food availability and child consumption of food and nutrients, including the directionality of the associations. Future analysis is warranted to examine predictors of home food availability over time such as family context (e.g. older siblings, maternal employment)^(48)^ and other household factors (e.g. socio-economic status, nutrition literacy) as well as examine the predictive power of home food availability on child consumption and subsequent child health outcomes. Third, the HFI was designed to capture an array of foods and beverages available in the home, but Fulkerson and colleagues note that the HFI is not an exhaustive inventory, nor does it indicate the quantity of items available^(13)^. For example, homes with one can of soda may receive the same score as those with ten cans of soda. Fourth, the Block FFQ is a commonly used tool to ascertain information about a child’s diet; however, because parents report their child’s intake of various foods, they can under- or overestimate consumption. This can become problematic, considering the Block FFQ does not ask parents to estimate serving sizes. Instead, Nutrition Quest estimates serving sizes based on national data from the NHANES. Related to the fourth limitation, these findings could be extended by identifying and examining dietary patterns, as this would offer additional information regarding the trade-offs between consuming a variety of food groups.

### Conclusion

By capitalising on longitudinal data, the current study contributes significantly to the literature by examining changes in home food availability over time and whether information about child diet can be ascertained through home food availability. On average, it appears that less nutritious foods become increasingly available as children age, and these changes could lead to potential deficiencies in food and nutrient intake. In the current study, the HFI^(13)^ has demonstrated evidence of validity and appears to be a valuable tool for determining some information about food and nutrient intake (i.e. at least one indicator of food or nutrient intake was significantly associated with the HFI) among children from 24 to 48 months of age, with many of the associations remaining consistent over time. Evidence of validity may be strengthened in future research by examining whether multiple dimensions of the home food environment, such as the combination of the physical and sociocultural characteristics (e.g. parent feeding practices, child eating habits), are predictive of subsequent child dietary intake and weight status. Future research targeting the built environment and participation in federal food assistance programmes is warranted to promote the availability of nutritious foods and beverages in the home.

## Supporting information

Barton et al. supplementary material Barton et al. supplementary material

## Supplementary material

For supplementary material accompanying this paper visit https://doi.org/10.1017/S1368980024000375
