# Longitudinal changes in home food availability and concurrent associations with food and nutrient intake among children at 24–48 months – CORRIGENDUM

**DOI:** 10.1017/S1368980026103103

**Published:** 2026-07-17

**Authors:** Jennifer M. Barton, Arden L. McMath, Stewart P. Montgomery, Sharon M. Donovan, Barbara H. Fiese

The authors apologise for the omission of full funding information of General Mills to Sharon M. Donovan and Naiman Khan. This has now been added.

## References

[ref1] Barton JM , McMath AL , Montgomery SP , Donovan SM , Fiese BH (2024) Longitudinal changes in home food availability and concurrent associations with food and nutrient intake among children at 24–48 months. Public Health Nutrition 27(1):e62. doi:10.1017/S1368980024000375 38305130 PMC10897571

