# Supplementary material for: Longitudinal changes in home food availability and concurrent associations with food and nutrient intake among children at 24–48 months
Source: Public Health Nutr. 2024 Feb 2;27(1):e62. doi: 10.1017/S1368980024000375 (PMC10897571; doi:10.1017/S1368980024000375)
Supplement: Barton et al. supplementary material [file S1368980024000375sup001.docx]

| Supplemental Table 1  *Descriptive statistics and adjusted Repeated Measures ANOVAs results for HFI category scores at 24, 36, and 48 months* | | | | | | | | | | | |  |  |  |
| --- | --- | --- | --- | --- | --- | --- | --- | --- | --- | --- | --- | --- | --- | --- |
| HFI Category |  | 24 months  (*n* = 413) | | 36 months  (*n* = 389) | | 48 months  (*n* = 370) | | Change over time | | Adjusted with Covariates | | | |  |
| *Total number of [items] in home* | Range | *M* | SD | *M* | SD | *M* | SD | *F* | *p*-value | *F* | *p*-value | | | |
| Fruits | 0–26 | 9.62 | 3.80 | 9.91 | 3.90 | 10.05 | 3.96 | *F* (2,743) = 1.57 | .209 | *F* (2, 515) = 0.17 | .845 | | | |
| Vegetables | 0–20 | 10.41^a^ | 3.48 | 10.63^a^ | 3.54 | 10.97^b^ | 3.59 | *F* (2, 741) = 3.93 | **.020** | *F* (2, 514) = 2.96 | .053 | | | |
| Vegetables – Excl. Potatoes | 0–9 | 9.64^a^ | 3.35 | 9.90^a^ | 3.38 | 10.22^b^ | 3.44 | *F* (2, 741) = 4.25 | **.015** | *F* (2, 514) = 3.38 | **.035** | | | |
| Dairy – Regular Fat | 0–9 | 4.31 | 1.67 | 4.34 | 1.75 | 4.41 | 1.73 | *F* (2, 745) = 0.47 | .627 | *F* (2, 516) = 0.07 | .929 | | | |
| Dairy – Reduced Fat | 0–12 | 3.13 | 1.88 | 3.17 | 1.74 | 3.05 | 1.87 | *F* (2,745) = 0.72 | .489 | *F* (2, 516) = 1.37 | .254 | | | |
| Whole Grains – Bread, WW Cereal | 0–6 | 2.00 | 1.27 | 1.93 | 1.19 | 1.97 | 1.20 | *F* (2, 742) = 0.83 | .438 | *F* (2, 514) = 0.80 | .451 | | | |
| Non-Whole Grains – Bread, HS Cereal | 0–8 | 3.74^a^ | 1.58 | 3.87^a^ | 1.65 | 4.11^b^ | 1.62 | *F* (2, 743) = 8.32 | **< .001** | *F* (2, 515) = 7.89 | **< .001** | | | |
| Processed Meats | 0–4 | 1.60^a^ | 1.06 | 1.66^a^ | 1.12 | 1.77^b^ | 1.08 | *F* (2,740) = 4.49 | **.012** | *F* (2, 513) = 2.25 | .107 | | | |
| Other Meats and Non-Dairy Proteins | 0–12 | 6.78 | 1.60 | 6.94 | 1.69 | 6.86 | 1.59 | *F* (2, 743) = 1.15 | .319 | *F* (2, 515) = 3.03 | **.049** | | | |
| Beverages – Regular Sugar | 0–6 | 2.03 | 1.24 | 1.99 | 1.20 | 2.02 | 1.28 | *F* (2,743) = 0.35 | .706 | *F* (2, 515) = 0.12 | .883 | | | |
| Candy | 0–5 | 2.09^a^ | 1.56 | 2.47^b^ | 1.59 | 2.60^bc^ | 1.62 | *F* (2, 741) = 27.45 | **< .001** | *F* (2, 514) = 14.62 | **< .001** | | | |
| Frozen Desserts | 0–3 | 0.78 | 0.69 | 0.80 | 0.62 | 0.89 | 0.64 | *F* (2, 741) = 2.97 | .052 | *F* (2, 515) = 1.48 | .229 | | | |
| Prepared Desserts | 0–6 | 1.26 | 1.04 | 1.33 | 1.06 | 1.28 | 1.04 | *F* (2, 743) = 0.48 | .618 | *F* (2. 515) = 0.76 | .470 | | | |
| Savory Snacks | 0–10 | 4.93^a^ | 1.87 | 5.08^a^ | 1.93 | 5.34^b^ | 1.90 | *F* (2, 743) = 8.14 | **< .001** | *F* (2, 513) = 4.45 | **.012** | | | |
| Microwavable/Quick-Cook Foods | 0–9 | 2.21^a^ | 1.67 | 2.44^b^ | 1.72 | 2.66^bc^ | 1.80 | *F* (2, 741) = 16.62 | **< .001** | *F* (2, 514) = 13.57 | **< .001** | | | |
| Obesogenic Score 1 | 0–57 | 28.73^a^ | 7.63 | 29.34^a^ | 7.71 | 30.35^b^ | 8.39 | *F* (2, 745) = 11.14 | **< .001** | *F* (2, 658) = 9.79 | **< .001** | | | |
| Obesogenic Score 2 | 0–54 | 26.87^a^ | 7.30 | 27.55^b^ | 7.36 | 28.48^c^ | 8.03 | *F* (2, 745) = 12.80 | **< .001** | *F* (2, 658) = 10.83 | **< .001** | | | |
| Obesogenic Score 3 | 0–52 | 24.41^a^ | 6.82 | 25.00^a^ | 6.92 | 26.02^b^ | 7.44 | *F* (2, 744) = 13.13 | **< .001** | *F* (2, 658) = 10.67 | **< .001** | | | |
| *Note*. Results from the adjusted Repeated Measures ANOVAs with covariates associated with missingness: monthly household income and maternal employment status and age at 6 weeks. Underlined *p*-values in the adjusted column differed from the unadjusted *p*-values; Vegetables were now marginally significant, Processed Meats were no longer significant, and Other Meats and Non-Dairy Proteins became significant. All HFI scores are calculated as sum scores based on the original instructions by Fulkerson and colleagues (2008). Frozen desserts, prepared desserts, and savory snacks only include “regular fat” items; “reduced fat” items were not included in those three scores. Two modifications were made for Whole Grains and Non-Whole Grains where whole wheat cereal and high sugar cereals were added to their respective categories.  Abbreviations: WW, whole wheat; HS, high sugar.  Post hoc pairwise comparisons with a Bonferroni correction were used. ^a-c^ Within a row, means without a common superscript differ at *p* < .05; superscripts refer to the unadjusted RMANOVAs. | | | | | | | | | | | | |  |  |
